# Association of Bitter Taste Receptor T2R38 Polymorphisms, Oral Microbiota, and Rheumatoid Arthritis

**DOI:** 10.3390/cimb43030103

**Published:** 2021-10-09

**Authors:** Vivianne Cruz de Jesus, Manu Singh, Robert J. Schroth, Prashen Chelikani, Carol A. Hitchon

**Affiliations:** 1 Manitoba Chemosensory Biology Research Group, Department of Oral Biology, Rady Faculty of Health Sciences, University of Manitoba, Winnipeg, MB R3E 0W4, Canada; cruzdejv@myumanitoba.ca (V.C.d.J.); robert.schroth@umanitoba.ca (R.J.S.); 2 Children’s Hospital Research Institute of Manitoba (CHRIM), Winnipeg, MB R3E 3P4, Canada; 3 Department of Biochemistry and Biomedical Sciences, McMaster University, Hamilton, ON L8S 4K1, Canada; singhm93@mcmaster.ca; 4 Department of Preventive Dental Science, Rady Faculty of Health Sciences, Dr. Gerald Niznick College of Dentistry, University of Manitoba, Winnipeg, MB R3E 0W4, Canada; 5 Department of Internal Medicine, Rady Faculty of Health Sciences, Max Rady College of Medicine, University of Manitoba, Winnipeg, MB R3A 1M4, Canada

**Keywords:** oral–systemic disease, taste genetics, autoimmune disease, rheumatoid arthritis, oral microbiome, G protein-coupled receptor

## Abstract

The association of taste genetics and the oral microbiome in autoimmune diseases such as rheumatoid arthritis (RA) has not been reported. We explored a novel oral mucosal innate immune pathway involving the bitter taste G protein-coupled receptor T2R38. This case–control study aimed to evaluate whether T2R38 polymorphisms associate with the buccal microbial composition in RA. Genomic DNA was obtained from buccal swabs of 35 RA patients and 64 non-RA controls. *TAS2R38* genotypes were determined by Sanger sequencing. The buccal microbiome was assessed by Illumina MiSeq sequencing of the V4-*16S rRNA* gene. Bacterial community differences were analyzed with alpha and beta diversity measures. Linear discriminant analysis effect size identified taxa discriminating between RA versus non-RA and across *TAS2R38* genotypes. *TAS2R38* genotype frequency was similar between RA and non-RA controls (PAV/PAV; PAV/AVI; AVI/AVI: RA 42.9%; 45.7%; 11.4% versus controls 32.8%; 48.4%; 18.8%, chi-square (2, N = 99) = 2.1, *p* = 0.35). The relative abundance of *Porphyromonas,* among others, differed between RA and non-RA controls. The relative abundance of several bacterial species also differed across *TAS2R38* genotypes. These findings suggest an association between T2R38 polymorphisms and RA buccal microbial composition. However, further research is needed to understand the impact of T2R38 in oral health and RA development.

## 1. Introduction

Recent evidence suggests an association between oral infections and systemic conditions, including rheumatoid arthritis (RA) [1]. RA and periodontal disease (PD), a leading cause of tooth loss, share similar phenotypes of inflammation causing the loss of adjacent calcified bone. RA is a chronic autoimmune disease characterized by joint inflammation that damages articular structures (bone and cartilage) leading to functional impairment. While the cause or trigger to develop RA remains unknown, genetic and environmental risks factors, including smoking and PD, are believed to play important roles. Periodontitis is a chronic inflammatory periodontal disease in which dysregulated immune responses against oral microbes lead to the loss of periodontal tissues and subsequently, tooth loss, if left untreated [2,3,4]. A strong and possibly bidirectional association between these conditions has been described. The molecular links between RA and oral diseases remain unclear but may involve oral microbial dysbiosis and altered host mucosal immunity [5,6].

Chemosensory bitter taste receptors (T2Rs) enable the sensation of bitter substances and have newly recognized roles as mediators of innate immunity. T2Rs are expresse d in many oral and extraoral tissues, including immune cells, and mediate host–pathogen interactions, suggesting relevance to both oral and systemic diseases [7,8,9,10,11,12,13,14]. T2R38 is the most studied taste receptor and it influences the ability of humans to taste both 6-n-propylthiouracil (PROP) and phenylthiocarbamide (PTC). There are three common single-nucleotide polymorphisms (SNPs, rs713598, rs1726866, and rs10246939) in the human T2R38-encoding gene (*TAS2R38*) that result in two common (AVI and PAV) and some rare (AAV, AAI, PAI, and PVI) haplotypes. The amino acid combination of proline, alanine, and valine at the amino acid positions 49, 262, and 296 results in the functional form of the receptor (PAV, taster), conferring the ability to taste PROP and PTC. Individuals with the non-functional AVI/AVI (alanine, valine, and isoleucine) genotype are not able to taste PROP and PTC and are referred to as non-tasters [15,16,17]. The PAV/PAV genotype (supertaster) has been linked to a higher expression of immunomodulating host defense peptides compared to the AVI/AVI (non-taster) genotype [18]. Supertasters are also less likely to develop chronic rhinosinusitis than non-tasters and have less bacterial biofilm formation [7,19,20,21,22]. The involvement of T2Rs in oral innate immune responses has only recently begun to be elucidated, and recent studies suggested that T2R-mediated antimicrobial responses play important roles in the maintenance of oral health [12,13,14].

The association of taste genetics and the oral microbiome in autoimmune rheumatic diseases such as RA has not been previously explored. Since periodontal disease, immune responses to oral pathogens, and autoimmunity can predate clinical RA [23], and since immune responses to oral pathogens associate with RA autoantibodies [24], we hypothesized that the greater innate immune response observed among people with the *TAS2R38* PAV/PAV genotype could lead to dysbiosis affecting key oral microbes implicated in periodontal disease and RA pathogenesis. In individuals predisposed to developing RA, T2R38-mediated oral inflammation could also lead to autoimmunity. The aim of this study was to characterize the composition of the buccal microbiome according to RA status and *TAS2R38* genotypes. We show evidence that polymorphisms in *TAS2R38* may affect the composition of the buccal microbiota in RA, providing further support for the role of oral mucosal immunity and host–microbial interactions contributing to the association between RA and oral health.

## 2. Materials and Methods

### 2.1. Study Population, Design, and Sample Collection

Adults with diagnosed RA [25] were recruited from rheumatology clinics in Winnipeg, Manitoba, Canada. People without RA or other chronic autoimmune conditions (non-RA controls) were recruited from family members or the community. Recruitment took place between January 2017 to February 2018 and sample size was based on convenience of recruitment. All participants completed questionnaires regarding general health, oral health, oral hygiene practices, and use of medication. Tender and swollen joint examinations were conducted by a rheumatologist. C-reactive protein (CRP) was measured by the local hospital laboratory. Circulating rheumatoid factor (RF measured by nephelometry) and anti-cyclical citrullinated peptide antibodies (ACPA, measured by CCP2 ELISA) were tested by the hospital laboratory and positivity determined according to the manufacturer’s guidelines. Buccal swab samples were collected by swabbing the inside of the subject’s inner cheek with a sterile swab and were stored at −80 °C for batch processing.

### 2.2. TAS2R38 Sequencing

Genomic DNA (gDNA) was extracted from buccal swabs using the QIAamp DNA mini kit (Qiagen, Hilden, Germany) following the manufacturer’s protocol. The paired-end Sanger sequencing of the *TAS2R38* gene, using the TAS2R38E01F (5′AGCTGGATGCTTTGTGAAGG 3′) and TAS2R38E01R (5′ TGAGTTCATTGATACAAAGTGGAGA 3′) primers, was conducted by McGill University—Génome Québec Innovation Centre (Montreal, QC, Canada). Sequences were compared to a reference sequence (NM_176817) using BLAST analysis (www.ncbi.nlm.nih.gov, accessed on 20 July 2018). The 3 most common *TAS2R38* variants (rs713598, rs1726866, and rs10246939) were analyzed to determine the participant’s haplotypes. Presence of G, T, and A at the 145, 785, and 886 nucleotide positions indicated the AVI haplotype, while C, C, and G indicated the PAV haplotype. The DNA sequences were analyzed with the Chromas v.2.6.6 software (Technelysium, South Brisbane, Australia).

### 2.3. 16S rRNA Amplicon Sequencing and Data Analysis

The same gDNA as above was used for *16S rRNA* gene sequencing to determine the buccal microbial community. Quality control, library preparation, and paired-end Illumina MiSeq PE250 sequencing of the V4 region of the *16S rRNA* gene using the 515F (5′-GTGCCAGCMGCCGCGGTAA-3′) and 806R (5′ -GGACTACHVGGGTWTCTAAT- 3′) primers were performed by McGill University—Génome Québec Innovation Centre (Montreal, QC, Canada). Sequencing data were analyzed using QIIME2 (Quantitative Insights into Microbial Ecology) version 2018.11 [26]. Demultiplexed and trimmed sequences were denoised and filtered with DADA2. Amplicon sequence variants (ASVs) were aligned with Mafft and Fasttree2 was used to make a phylogeny tree [27]. Taxonomic assignment of ASVs was performed with the classify-sklearn naïve Bayes taxonomy classifier against the *e*HOMD database (HOMD, version 15.1, Cambridge, MA, USA) [26,28,29,30]. Only taxa with non-zero counts in at least 5% of the samples were retained.

### 2.4. Statistical Analysis

Descriptive statistics compared diagnostic groups using chi-square, Fisher’s exact, Mann–Whitney U, or Kruskal–Wallis tests as appropriate. Associations between *TAS2R38* haplotype and diagnosis were tested in genotypic, dominant, and recessive models. Microbiome alpha diversity (within samples) was calculated using the Shannon diversity index within QIIME2. Beta diversity (between samples) was evaluated using weighted UniFrac distances. Larger UniFrac distances indicate greater differences in bacterial community composition between groups. The significance of differences in buccal bacterial diversity by RA status or by *TAS2R38* genotype was determined using the permutational analysis of variance (PERMANOVA) test with 999 permutations. Microbial grouping was visualized using principle coordinate analysis (PCoA) and the R “ggplot2” package [31]. Linear discriminant analysis (LDA) effect size (LefSe) analysis (available at http://huttenhower.sph.harvard.edu/galaxy, accessed on 4 October 2021) with an alpha value of <0.05 was performed to identify the taxa that better discriminated between the groups. The LDA score grades the taxa according to their importance and only taxa with an LDA score >2.0 were plotted [32]. A *p* ≤ 0.05 was considered to be statistically significant. The statistical tests were performed in R (version 3.5.2).

The study was conducted according to the guidelines of the Declaration of Helsinki. The study protocol was approved by the University of Manitoba’s Health Research Ethics Board (HREB # HS15191—B2001:070). All participants provided informed written consent to participate in the study, including limited chart review and the collection of oral samples.

## 3. Results

### 3.1. Participants

Thirty-five patients with diagnosed RA and sixty-four non-RA controls, of whom 58 (91%) were first-degree relatives at risk for future RA, had *TAS2R38* genotyping and microbiome analysis performed. RA patients were older than non-RA controls (RA 51.2 ± 13.8 years versus non-RA controls 43.3 ± 11.5 years, *p* < 0.01) and more likely to be female (RA 83% versus non-RA controls 50%, *p* < 0.01). There were no differences in general health, smoking, or self-reported oral health symptoms and habits between the RA and non-RA groups (Table 1). RA patients had low disease activity. Overall, we detected a high prevalence of smoking and poor oral health habits in our cohort.

### 3.2. Taste Genetics and RA

The population distribution of AVI and PAV alleles was in Hardy–Weinberg equilibrium. No significant differences were seen across the genotypes between RA and controls in genotype or dominant models after controlling for age and sex (PAV/PAV; PAV/AVI; AVI/AVI: RA 42.9%; 45.7%; 11.4% versus controls 32.8%; 48.4%; 18.8%, chi-square (2, *N* = 99) = 2.1, *p* = 0.35). Since 91% of the controls were relatives of the RA participants and thus likely to have a similar genetic background, we compared the TAS2R38 genotype frequencies of the RA patients with a published North American control population [19]. This population-based study was selected due to the fact that it has a large control group (*N* = 347; PAV/PAV 20%, PAV/AVI 51%, AVI/AVI 29%) from a similar region (North America) as our RA population and provided the frequencies of TAS2R38 genotypes among the groups, not only haplotypes. There was a significant difference in genotype distribution between RA and this control population (chi-square (2, *N* = 382) = 11.1 *p* = 0.004). Similar results were observed when using PAV-recessive (*p* = 0.002) and PAV-dominant models (*p* = 0.03). The attributable risk of the PAV/PAV genotype to RA over the North American control population was 8% (1% over the local controls). Most demographic and clinical features were similar across TAS2R38 genotypes (Table 2).

### 3.3. The Oral Microbiome, TAS2R38 Genotypes, and RA

**Figure 1:**
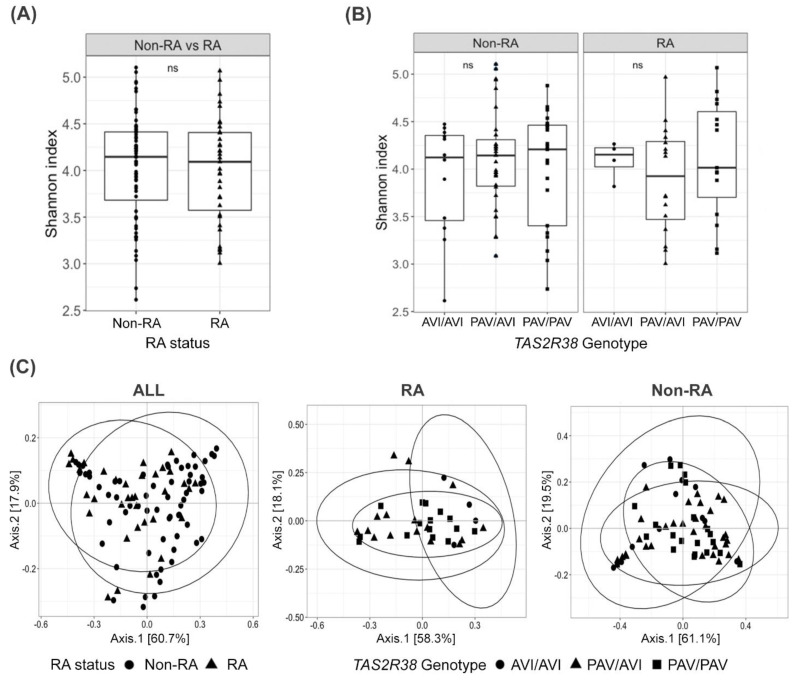
Bacterial diversity of buccal samples from RA patients and non-RA controls according to RA status and *TAS2R38* genotypes. (**A**) Alpha diversity Shannon index between the RA and non-RA groups and (**B**) among individuals with different *TAS2R38* genotypes. No significant difference was observed in Shannon index between the groups (ns, *p* > 0.05). (**C**) Beta diversity, principle coordinate analysis (PCoA) of weighted UniFrac distances. In the PCoA plot, each data point represents one sample, and they are shaped according to RA status and the participant’s *TAS2R38* genotypes. The 95% confidence ellipse plots are shown for each group. Beta diversity analysis revealed significant differences in the buccal bacterial composition between RA and non-RA controls (*p* = 0.02) but not between TAS2R38 genotypes (*p* > 0.05). RA, rheumatoid arthritis; PAV, T2R38 functional allele with the amino acids proline, alanine, and valine; AVI, T2R38 non-functional allele with alanine, valine, and isoleucine.

We investigated the buccal bacteriome according to RA status and *TAS2R38* genotype. Alpha diversity analysis showed a similar Shannon diversity index of bacterial species among RA compared to non-RA controls (*p* > 0.05) (Figure 1A). Beta diversity analysis revealed significant differences in the buccal bacteriome composition between RA and controls (*p* = 0.02, PERMANOVA, Figure 1C). No differences in alpha or beta diversity were seen by *TAS2R38* genotype (Figure 1B,C).

**Figure 2:**
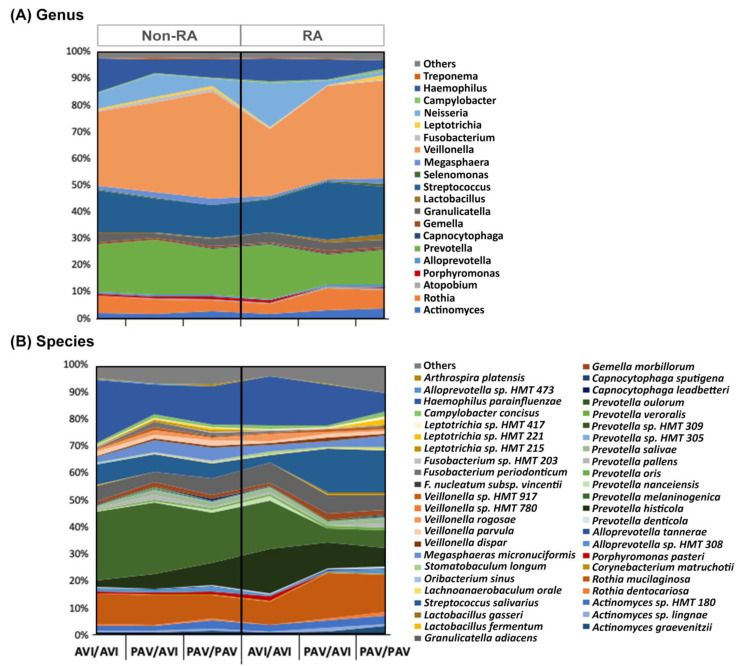
Taxonomic profile of the buccal bacteriome according to RA status and *TAS2R38* genotypes. Relative abundance of bacterial (**A**) genera and (**B**) species. Colors were only assigned to 25% of the most abundant genera and species. RA, rheumatoid arthritis; HMT, human oral taxon; PAV, T2R38 functional allele with the amino acids proline, alanine, and valine; AVI, T2R38 non-functional allele with alanine, valine, and isoleucine.

Figure 2 shows the taxonomic profile of the buccal samples according to RA status and *TAS2R38* genotype. We identified 11 phyla, 128 genera, and 315 bacterial species. The most abundant bacterial genera in our cohort were *Veillonella*, *Prevotella*, and *Streptococcus* (Figure 2).

**Figure 3:**
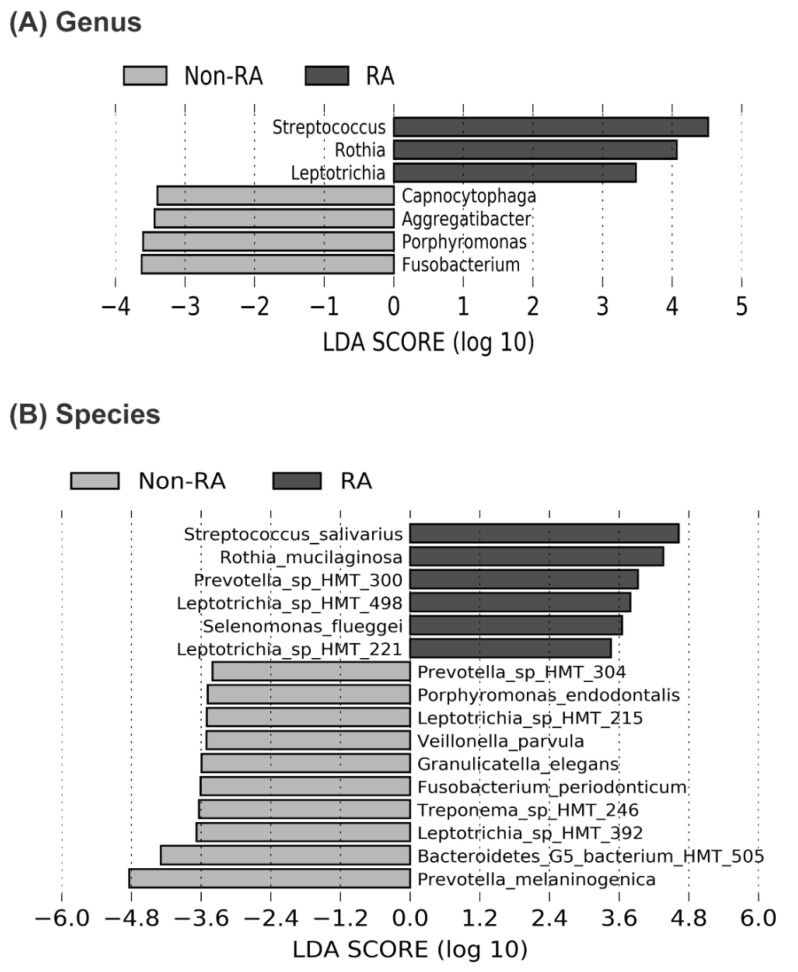
Most highly differentially abundant bacteria in buccal swab samples from patients with RA and non-RA control individuals by the LEfSE (linear discriminant analysis effect size) analysis. The graph demonstrates log10-fold change for the most highly enriched bacterial (**A**) genera and (**B**) species in RA (dark gray bars) and non-RA controls (light gray bars). All bacterial species and genera listed showed an adjusted *p*-value < 0.05. RA, rheumatoid arthritis; HMT, human oral taxon.

**Figure 4:**
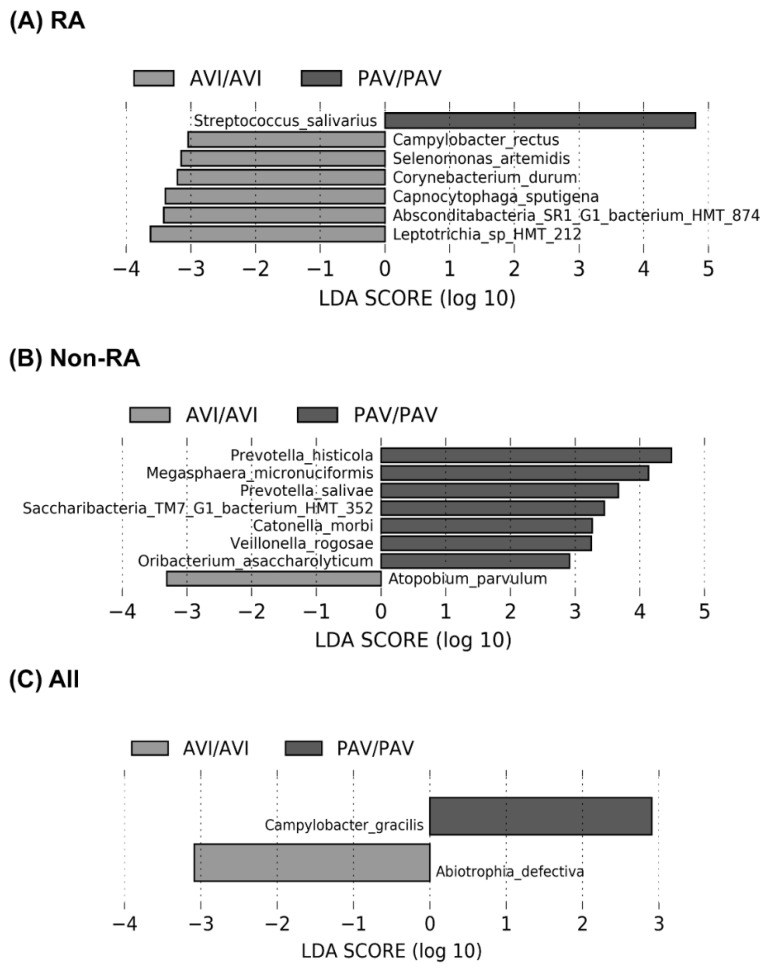
Most highly differentially abundant bacteria in buccal swab samples from participants with the homozygous AVI/AVI and PAV/PAV *TAS2R38* genotypes by the LEfSE (linear discriminant analysis effect size) analysis. Species identified as discriminating features for PAV/PAV versus AVI/AVI genotypes among (**A**) RA patients (*N = 35*), (**B**) non-RA controls (*N = 64*), and (**C**) all study participants (*N = 99*). All bacterial species and genera listed showed an adjusted *p*-value < 0.05. RA, rheumatoid arthritis; HMT, human oral taxon; PAV, T2R38 functional allele with the amino acids proline, alanine, and valine; AVI, T2R38 non-functional allele with alanine, valine, and isoleucine.

Linear discriminant analysis (LDA) effect size (LEfSe) analysis demonstrated differences in the relative abundance of some taxa between RA and non-RA controls. At the genus level, *Streptococcus, Rothia*, and *Leptotrichia* were most abundant in RA, whereas *Fusobacterium, Porphyromonas, Aggregatibacter*, and *Capnocytophaga* were relatively enriched in non-RA (Figure 3A). At the species level, *Streptococcus salivarius, Rothia mucilaginosa, Prevotella sp., Leptotrichia sp.,* and *Selenomonas fueggei* were more abundant in RA, whereas other Prevotella species, including *Prevotella melaninogenica, Bacteroidetes, Fusobacterium periodonticum, Granulicatella elegans,* and *Porphyromonas endodontalis,* among others, were abundant in non-RA controls (Figure 3B). Upon subgroup analysis, differences in bacterial relative abundances across *TAS2R38* genotypes were also observed within the RA group (Figure 4A) and non-RA controls (Figure 4B). The buccal microbial composition also differed between individuals with the homozygous PAV/PAV (“supertasters”) and those carrying the AVI/AVI genotype (“non-tasters”), regardless of their RA status (Figure 4C). Within the RA group, the *Streptococcus salivarius* was more abundant in the supertaster PAV/PAV subgroup compared to the non-taster AVI/AVI.

## 4. Discussion

In this study, we investigated whether a novel mucosal innate immune pathway mediated by bitter taste receptor T2R38 contributes to the pathobiology linking RA and oral health. Our findings show an over-representation of the functional PAV/PAV genotype in people with RA compared to the literature population controls, and that the composition of the RA buccal bacteriome, which differs from non-RA controls, also differs by *TAS2R38* genotype. These findings support a role for oral dysbiosis and mucosal innate immunity involving T2Rs in RA.

To our knowledge, *TAS2R38* genotyping has not been studied in autoimmune rheumatic disease. T2R38 is the most studied T2R as it affects the ability to taste PROP and PTC. However, the sensitivity to PROP and PTC changes with age and dietary and oral habits, such as smoking, and varies by method used for sensory analysis. Thus, T2R association studies relying on PROP/PTC sensory analysis must be interpreted with some caution. We determined the TAS2R38 genotypes with gene sequencing. The *TAS2R38* PAV/PAV genotype frequency observed in our RA population was higher than that reported in previous studies of chronic airway infection in North American populations (i.e., rhinosinusitis and cystic fibrosis) [19,22] and in the known population genotype distribution of predominantly European descent [15,33]. We attributed this to an association with RA. However, global variation in *TAS2R38* genotype distribution exists and our region has a high proportion of North American Indigenous people who are known to have a high prevalence of the PAV/PAV haplotype [15] and also severe RA [34]. We did not see a significant difference in *TAS2R38* haplotypes between RA and regional non-RA controls, possibly due to the small sample size or due the fact that many of the control subjects were first-degree relatives of the RA patients, and thus were likely to share similar genotypes. These relatives represent a group at risk for developing future RA.

Since genetic variations in *TAS2R38* have functional consequences that, in addition to taste perception, may contribute to antimicrobial responses [18,20], we investigated the buccal microbiome in our RA and non-RA control subjects, many of whom were at risk for developing future RA, and compared the microbiome across *TAS2R38* genotypes. Our findings are consistent with other reports finding the enrichment of periodontal pathogens in oral, pulmonary, and gastrointestinal microbiomes obtained from individuals at risk for RA [35,36,37,38,39,40]. Relative depletion of *P. gingivalis* has been reported in RA patients compared to anti-citrullinated protein autoantibody (ACPA)-positive individuals who are at risk for developing future RA [38]. Regional differences in diet, habits, and oral health may contribute to the variability seen across microbiome studies in RA.

T2R-mediated antimicrobial responses are important to maintaining oral health [12,13,14]. However, dysregulated oral innate immunity can lead to dysbiosis and may contribute to oral and systemic diseases and to autoimmunity. The development of autoantibodies, in particular ACPAs, is a key feature of rheumatoid arthritis. ACPAs can predate RA onset by several years [41]. ACPAs are also found in individuals with severe periodontitis, and key periodontal pathogens, *P. gingivalis* and *Aggregatibacter actinomycetemcomitans,* are capable of citrullinating host tissue. *P. gingivalis* is the only known prokaryote expressing peptidylarginine deiminase (PPAD), the enzyme responsible for citrullination. *A. actinomycetemcomitans* secretes leukotoxin A, a pore-forming toxin that induces neutrophil death by leukotoxic hypercitrullination [42,43]. Recent reports showed a high burden of PD in ACPA-positive subjects at risk for future RA, and decreased abundance of *P. gingivalis* in newly onset RA with PD [44,45]. Here, we found that *Porphyromonas* and *Aggregatibacter* were depleted in RA compared to at-risk controls, although we were unable to specifically identify *P. gingivalis* or *A. actinomycetemcomitans* [43,46] due to low counts. Similarly, we did not see robust associations between T2R genotype and circulating ACPA, probably due to the small sample size. However, we and others have previously shown that ACPA levels associate with increased titers of antibodies to *P. gingivalis* in people at risk for RA [24,47], as do antibodies to *Prevotella* in RA [48], and that antibody titers to leukotoxin A are increased in ACPA-positive at-risk individuals [43,49]. Effective anti-*Porphyromonas* immune responses in pre-clinical RA could explain the observed depletion of *Porphyromonas* once RA is established, especially in people carrying the functional PAV allele, whereas persistent periodontal inflammation could contribute to the persistent elevated ACPA responses seen in RA with periodontal disease, and possibly RA disease activity seen with periodontal disease. Interestingly, it has been reported that non-surgical oral interventions that improved both periodontal disease and arthritis also reduced the abundance of select oral pathogens [50]. Overall, these data suggest immune targeting of periodontal pathogens may contribute to RA pathology and that T2R38-mediated antibacterial responses may play a role.

Our microbiome findings are based on samples from the buccal mucosa which, while reflective of overall oral health, may not adequately assess microbiota relevant for PD; the microbiota of subgingival samples better reflects periodontal status. In addition, while the prevalence of reported problematic oral health habits and symptoms was similarly high across groups in our study, symptoms do not adequately predict oral disease and we lack formal periodontal exams. We also did not fully assess diet, nutraceutical use, or other geographic or cultural factors that may affect the oral microbiome. However, these are likely similar given that many of the non-RA controls were relatives of the RA subjects. Our sample size is small and may not be representative of other populations. In addition, we studied only the predominant haplotypes of T2R38. Other T2R polymorphisms may be important for RA.

In this study, we show evidence that polymorphisms in *TAS2R38* may be associated with differences in the microbial composition of the buccal mucosa in RA. However, replication in larger populations with paired periodontal exams and biosamples are needed to confirm the associations described.

## Figures and Tables

**Table 1:** Characteristics of the study population.

Characteristic	Non-RA (*n* = 64)	RA (*n* = 35)	*p*-Value ^a^
**Demographics**			
Age, mean ± SD	43.3 ± 11.53	51.2 ± 13.8	**0.004**
Sex, female	32 (50.0%)	29 (82.9%)	**0.001**
**RA features**			
ACPA positive ^b^	2 (3.4%)	23 (79.3%)	**<0.001**
ACPA titer if positive ^b,^^c^	155 (19,155)	300 (32.5, 300)	0.35
RF positive ^b^	8 (13.6%)	22 (76.9%%)	**<0.001**
RF titer if positive ^b^	37 (22.3, 50.1)	382 (172, 1047.8)	**<0.001**
Current DMARD	NA	21 (65.6%)	**-**
Swollen joint count	NA	1.0 (0.0, 3.3)	**-**
CRP (mg/L)	2.0 (1.0, 4.7)	6.0 (1.0, 15.0)	**0.02**
**Overall health**			
Current smoking	47 (79.7%)	21 (75.0%)	0.62
Diabetes mellitus	14 (23.3%)	5 (15.2%)	0.35
BMI	28.6 (25.5, 33.0)	28.23 (24.3, 32.8)	0.75
Currently on antibiotics	1 (1.7%)	3 (10.7%)	0.06
**Oral Health**			
Denture	14 (23.7%)	10 (33.3%)	0.33
Gum bleeding			0.17
Never	15 (27.8%)	11 (50.0%)	
Rarely	20 (37.0%)	5 (22.7%)	
Sometimes/often/always	19 (35.2%)	6 (27.3%)	
Metallic taste			0.91
Never	37 (68.5%)	14 (63.6%)	
Rarely	6 (11.1%)	3 (13.6%)	
Sometimes/often/always	11 (20.4%)	5 (22.7%)	
Tender or painful gum			0.31
Never	27 (50.0%)	10 (45.5%)	
Rarely	9 (16.7%)	7 (31.8%)	
Sometimes/often/always	18 (33.3%)	5 (22.7%)	
Feeling of loose teeth			0.95
Never	38 (70.4%)	15 (68.2%)	
Rarely	10 (18.5%)	4 (18.2%)	
Sometimes/often/always	6 (11.1%)	3 (13.6%)	
Brush/week			0.44
Less than 6 times	34 (60.7%)	13 (50.0%)	
7 to 14 times	18 (32.1%)	9 (34.6%)	
More than 15 times	4 (7.1%)	4 (15.4%)	
Floss/week			0.31
Less than 6 times	47 (83.9%)	18 (69.2%)	
7 to 14 times	7 (12.5%)	6 (23.1%)	
15+ times	2 (3.6%)	2 (7.7%)	
Dentist visit			0.24
Never	7 (12.3%)	16 (59.3%)	
1–2 times per year	44 (77.2%)	16 (59.3%)	
2+ times per year	6 (10.5%)	5 (18.5%)	

Categorical variables are reported as n (%), continuous variables are median (25%, 75%), unless otherwise specified. Bold values indicate *p* ≤ 0.05. RA, rheumatoid arthritis; ACPA, anticitrullinated peptide antibodies; RF, rheumatoid factor; DMARD, disease-modifying antirheumatic drugs; CRP, C-reactive protein; BMI, body mass index; NA, not applicable; -, no statistical analysis performed. ^a^ Mann–Whitney U test for continuous variables; chi-square or Fisher-exact test for categorical variables. ^b^ Participants with ACPA ≥ 8 were considered ACPA positive, RF titer ≥ 20 was considered RF positive. ^c^ ACPA titer range for non-RA. ACPA and RF missing for 5 non-RA and 6 RA.

**Table 2:** Characteristics of the study population according to *TAS2R38* genotypes.

	Non-Taster AVI/AVI (*n* = 16)	Taster PAV/AVI (*n* = 47)	Super Taster PAV/PAV (*n* = 36)	*p*-Value ^a^
**Demographics**				
Age, mean ± SD	44.4 ± 11.1	44.7 ± 13.2	48.8 ± 13.1	0.35
Sex, female	12 (75.0%)	24 (54.5%)	25 (71.4%)	0.18
**Clinical features**				
RA	4 (25.0%)	16 (34.0%)	15 (41.7%)	0.49
ACPA positive ^b^	2 (13.3%)	11 (27.5%)	12 (35.4%)	0.26
ACPA titer if positive	82 (-)	223 (22.5, 300)	300 (885, 300.0)	0.34
RF positive ^b^	4 (26.7%)	11 (27.5%)	15 (45.5%)	0.21
RF titer if positive	243 (38, 243)	208 (56.4,4525)	109 (26.7, 1010)	0.9
Current DMARD	4 (26.7%)	7 (18.42%)	10 (32.3%)	0.41
**Overall health**				
Current smoking	10 (66.7%)	30 (76.9%)	28 (84.8%)	0.36
Diabetes mellitus	3 (20.0%)	6 (13.6%)	10 (29.4%)	0.23
BMI	31.8 (27.8, 36.6)	27.1 (23.3, 32.3)	28.2 (24.8, 31.3)	0.11
Currently on antibiotics	1 (6.7%)	1 (2.6%)	2 (6.2%)	0.70

Categorical variables are reported as n (%), continuous variables are median (25%, 75%), unless otherwise specified. RA, rheumatoid arthritis; ACPA, anticitrullinated peptide antibodies; RF, rheumatoid factor; CRP, C-reactive protein; DMARD, disease-modifying antirheumatic drugs; BMI, body mass index; AVI, T2R38 non-functional allele with alanine, valine, and isoleucine; PAV, T2R38 functional allele with proline, alanine, and valine. ^a^ From Kruskal–Wallis test with Bonferroni correction for continuous variables and chi-square test or Fisher’s exact test for categorical variables. ^b^ Participants with ACPA ≥ 8 were considered ACPA positive, RF titer ≥ 20 was considered RF positive, ACPA and RF missing for 1 AVI/AVI, 7 PAV/AVI and 3 PAV/PAV.

## Data Availability

Microbiome data are available for review upon reasonable request to the corresponding authors. Secondary data analysis is not permitted, and clinical and genetic data are not available due to participant consent and institutional ethics requirements.
